# Fully automatic prognostic biomarker extraction from metastatic prostate lesion segmentations in whole-body [^68^Ga]Ga-PSMA-11 PET/CT images

**DOI:** 10.1007/s00259-022-05927-1

**Published:** 2022-08-17

**Authors:** Jake Kendrick, Roslyn J. Francis, Ghulam Mubashar Hassan, Pejman Rowshanfarzad, Jeremy S. L. Ong, Martin A. Ebert

**Affiliations:** 1 School of Physics, Mathematics and Computing, University of Western Australia, Perth, WA Australia; 2 Medical School, University of Western Australia, Crawley, WA Australia; 3 Department of Nuclear Medicine, Sir Charles Gairdner Hospital, Perth, WA Australia; 4 Department of Nuclear Medicine, Fiona Stanley Hospital, Murdoch, WA Australia; 5 Department of Radiation Oncology, Sir Charles Gairdner Hospital, Perth, WA Australia; 6 5D Clinics, Claremont, WA Australia

**Keywords:** PSMA, PET/CT, Segmentation, Prognostic biomarkers, Deep learning, Prostate cancer

## Abstract

**Purpose:**

This study aimed to develop and assess an automated segmentation framework based on deep learning for metastatic prostate cancer (mPCa) lesions in whole-body [^68^Ga]Ga-PSMA-11 PET/CT images for the purpose of extracting patient-level prognostic biomarkers.

**Methods:**

Three hundred thirty-seven [^68^Ga]Ga-PSMA-11 PET/CT images were retrieved from a cohort of biochemically recurrent PCa patients. A fully 3D convolutional neural network (CNN) is proposed which is based on the self-configuring nnU-Net framework, and was trained on a subset of these scans, with an independent test set reserved for model evaluation. Voxel-level segmentation results were assessed using the dice similarity coefficient (DSC), positive predictive value (PPV), and sensitivity. Sensitivity and PPV were calculated to assess lesion level detection; patient-level classification results were assessed by the accuracy, PPV, and sensitivity. Whole-body biomarkers total lesional volume (TLV_auto_) and total lesional uptake (TLU_auto_) were calculated from the automated segmentations, and Kaplan–Meier analysis was used to assess biomarker relationship with patient overall survival.

**Results:**

At the patient level, the accuracy, sensitivity, and PPV were all > 90%, with the best metric being the PPV (97.2%). PPV and sensitivity at the lesion level were 88.2% and 73.0%, respectively. DSC and PPV measured at the voxel level performed within measured inter-observer variability (DSC, median = 50.7% vs. second observer = 32%, *p* = 0.012; PPV, median = 64.9% vs. second observer = 25.7%, *p* < 0.005). Kaplan–Meier analysis of TLV_auto_ and TLU_auto_ showed they were significantly associated with patient overall survival (both *p* < 0.005).

**Conclusion:**

The fully automated assessment of whole-body [^68^Ga]Ga-PSMA-11 PET/CT images using deep learning shows significant promise, yielding accurate scan classification, voxel-level segmentations within inter-observer variability, and potentially clinically useful prognostic biomarkers associated with patient overall survival.

**Trial registration:**

This study was registered with the Australian New Zealand Clinical Trials Registry (ACTRN12615000608561) on 11 June 2015.

## Introduction

Prostate cancer (PCa) is one of the most commonly diagnosed malignancies and a leading cause of cancer mortality in men throughout the world [1]. Detection of the disease in the early stages when it is confined to the prostate enables treatment with curative intent, typically in the form of radiotherapy or radical prostatectomy (RP), and is associated with high 5-year patient survival rates. Biochemical recurrence (BCR) following localised disease treatment is not uncommon, however, occurring in around 30% of patients and potentially leading to subsequent metastatic disease [2]. Patients with metastatic PCa (mPCa) have a considerably worse prognosis, with 5-year survival rates dropping substantially to 35% or less depending on the sites of involvement (e.g., lymph nodes and bone) [3, 4]. The extent and location of metastatic disease has implications for patient treatment considerations; therefore, the detection and localisation of mPCa lesions is clinically important [5].

Prostate-specific membrane antigen (PSMA) is a transmembrane protein that is significantly over-expressed in malignant prostate tissues [6]. This over-expression combined with the internalisation of the PSMA protein yields enhanced radioligand retention within tumour cells, thus making PSMA a promising molecular target for both diagnostic and therapeutic purposes [7]. These desirable properties of the PSMA receptor have driven the development of numerous small molecule ligands that bind with high affinity to the extracellular domain of PSMA [8]. These ligands can be labelled with positron-emitting isotopes such as ^68^Ga or ^18^F for diagnostic assessment of patients and are FDA-approved for prostate cancer imaging. Positron emission tomography (PET) imaging radioligands targeting PSMA have quickly become the standard of care diagnostic tool in managing biochemically recurrent PCa, demonstrating superiority to traditional imaging techniques such as bone scintigraphy with a computed tomography (CT) scan [9–11]. Theranostic pairing with therapeutic radioisotopes such as ^177^Lu is also possible, with high level evidence of clinical benefit in advanced metastatic disease [12, 13].

Detection and localisation of mPCa lesions is a prerequisite for targeted treatment procedures such as radiotherapy. PET imaging also facilitates the extraction of quantitative imaging biomarkers related to PSMA expression including standardised uptake value (SUV)_max_ of tumours and total scan SUV (SUV_total_) which may have prognostic potential. Precise disease localisation also plays an integral role in the burgeoning field of radiomics, whereby high volumes of imaging features are extracted from medical images to quantify tumour characteristics and inform a precision-medicine approach to patient management [14–16]. The radiomics approach has already demonstrated significant potential in the detection of mPCa lesions, prediction of future metastases development, and has yielded novel biomarkers that have been shown to correlate with overall survival (OS) in advanced PCa patients [17–19]. Segmentation of lesions, when done manually, has well documented limitations such as having a high labour burden (especially true for patients with a high volume of disease) as well as being subject to inter- and intra-observer variability [20, 21]. Fully automated detection and segmentation of disease, therefore, is highly desirable both in reducing manual user input and as a method to expedite quantitative feature extraction geared towards advancing more personalised patient interventions.

The use of artificial intelligence techniques to solve clinical problems has catapulted to the forefront of medical research in recent times [22]. Deep learning, in particular, has garnered significant attention as a method of abstracting high-level feature representations of input data, such as medical images, and learning the most salient features in a hierarchical manner. Fully convolutional networks (FCNs) such as the U-Net and its associated alternatives are capable of performing fully automatic segmentations of input images through the use of an encoder-decoder network architecture, where the encoder progressively down-samples input images into increasingly higher-level salient feature abstractions, and the symmetric decoder up-samples the generated feature maps to output the final semantic segmentation in the same resolution as the input image [23–25]. FCNs have been applied with great success in a number of biomedical contexts, demonstrating the ability to segment a wide variety of clinically relevant anatomical and physiological structures such as the prostatic gross tumour volume in PSMA PET images, whole-body multiple myeloma lesions on ^68^Ga-Pentifaxor PET/CT scans, and glioma brain tumours on ^18^F-fluoro-ethyl-tyrosine (FET) PET imaging [26–28]. A persistent challenge in the implementation of these networks, however, are the architectural, training, and image processing pipeline design choices that typically require both significant domain expertise and lengthy trial and error processes to configure optimally [29]. To mitigate this extensive trial and error process, Isensee et al. [30] developed nnU-Net, a self-configuring biomedical image segmentation framework that automates key aspects of the segmentation pipeline according to a set of formulated heuristics that are task-agnostic. The nnU-Net has demonstrated considerable generalisable potential by achieving state-of-the-art results across a wide variety of different biomedical image segmentation tasks [30].

Automated whole-body segmentation of mPCa lesions, which can be numerous and highly heterogeneous in size, shape, and anatomical location, is a challenging task. Proof-of-concept studies restricting their analysis to the segmentation of mPCa lesions in the pelvic area exist for [^68^Ga]Ga-PSMA PET/CT, [^18^F]F-PSMA PET/CT and multiparametric magnetic resonance imaging (mpMRI) modalities, and semi-automated approaches have been demonstrated for whole-body scan analysis [31–35]. In the present work, a fully automated mPCa lesion detection and segmentation tool is developed and evaluated for whole-body [^68^Ga]Ga-PSMA-11 PET/CT scans utilising the nnU-Net framework [30]. Model performance is assessed at the patient, lesion and voxel levels, and global biomarkers calculated from the automated segmentations are assessed for their potential to stratify mPCa patients based on overall survival (OS).

## Methods

### Patient cohort

A cohort of 193 patients with biochemically recurrent PCa following definitive treatment for localised prostate carcinoma who underwent imaging at Sir Charles Gairdner Hospital (SCGH) as part of a prospective trial registered with the Australian New Zealand Clinical Trials Registry (ACTRN12615000608561) were used in this study [10]. Biochemical recurrence was defined as either: (i) PSA levels greater than 0.2 ng/mL measured at least 6 weeks post radical prostatectomy, or (ii) PSA level 2 ng/mL above the previous PSA nadir measured at least 3 months post external beam radiotherapy. Patients were included if they showed either negative or oligometastatic disease (maximum of three lesions) on bone scintigraphy and abdominal CT staging scans. All patients received an initial baseline [^68^Ga]Ga-PSMA-11 PET/CT scan and then, if clinically indicated, a second follow-up scan approximately 6 months later, yielding a total of 337 scans available for use in the study. The decision to undertake follow-up imaging was made at the discretion of the treating physician to assess for sites of disease following treatment. Patients underwent therapy following their baseline scan according to standard clinical care, which could include radiotherapy to the prostatic bed, regional nodes or bone metastases, further surgery, systemic treatment in the form of chemotherapy or androgen deprivation therapy (ADT), or active surveillance. Appropriate ethics approval was obtained from the SCGH Human Research Ethics Committee (RGS1736).

### Imaging acquisition

The [^68^Ga]Ga-PSMA-11 PET/CT scans were obtained for all patients concurrently on a Siemens Biograph mCT 64 PET/CT scanner (CTI Inc, Knoxville, TN). Prior to acquisition, patients were asked to void their bladders. 2 MBq/kg of [^68^Ga]Ga-PSMA-11 was administered intravenously through a peripheral intravenous cannula as a slow push. PET/CT image acquisition began approximately 60 min after radiotracer administration. A low-dose CT (50 mAs, 120 kVp) from the middle of the thigh to the vertex of the skull was acquired first for attenuation correction, with PET emission data being acquired immediately after to ensure identical field of view. PET images were reconstructed with a voxel resolution of 4.07 × 4.07 × 2 mm^3^, while CT reconstructed voxel resolutions varied between 0.98 × 0.98 × 2 mm^3^ and 1.52 × 1.52 × 5 mm^3^.

### Ground truth definition

**Fig. 1:**
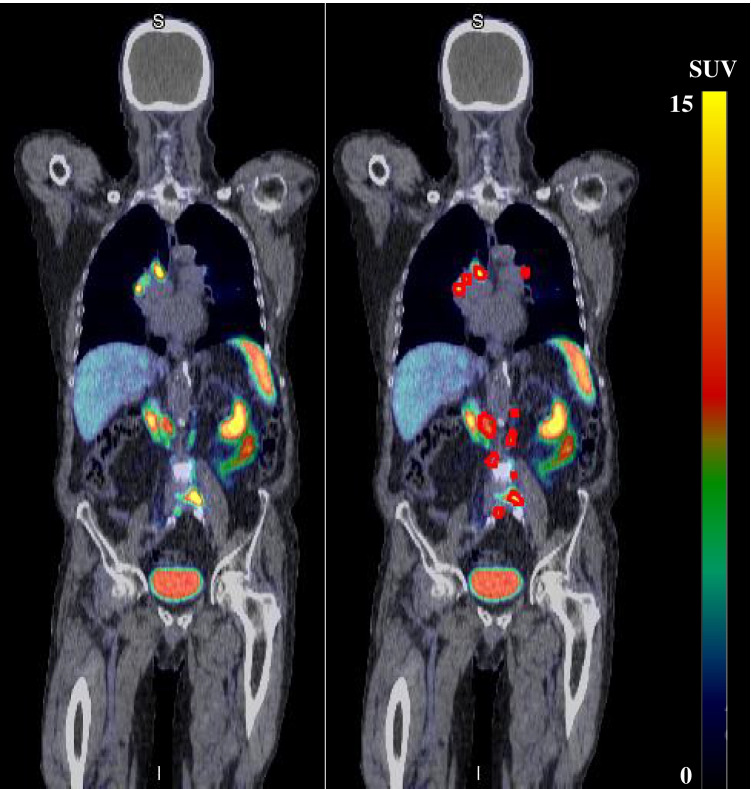
Exemplar whole-body [^68^Ga]Ga-PSMA PET/CT fusion scan acquired from the middle of the thigh to the skull vertex for a single patient. Screenshots were extracted from the MIM Encore software used for delineation and visualisation. On the left, a coronal slice from the PET/CT fusion is shown without any contouring, and on the right the same coronal slice is displayed with metastatic PCa lesions delineated clearly in red

Lesions for each patient scan were manually delineated by an expert Nuclear Medicine Physician (J.O.) using the MIM Encore radiation oncology software (MIM Software Inc., Cleveland, OH, USA) which were outputted as DICOM structure sets for subsequent use in the deep learning method. Areas of elevated tracer uptake were interpreted as lesions if they were deemed to be probably or definitely positive based on the 5-point scoring system detailed in published E-PSMA guidelines [36]. The segmentation process began with the application of a global SUV_bw_ > 3 threshold to the PET scan. Following this, included areas of physiologic uptake were manually discarded and any missed lesions were manually contoured, creating the final ground truth contour. Missed lesions needed to be added in about half of patient scans. Figure 1 shows a representative example of a [^68^Ga]Ga-PSMA-11 PET/CT scan with ground truth contours. Additionally, a random subset of scans underwent segmentation by a second independent observer (R.F., *n* = 28 scans) using the same segmentation methodology described above, allowing a quantification of inter-observer variability.

### Model training

Before training the model, PSMA-negative patient scans (*n* = 53) were separated from the total dataset and reserved solely as negative controls for model testing to mitigate the already large class imbalance in the dataset. Of the remaining PSMA-positive scans (*n* = 284), approximately 25% (*n* = 75) were randomly assigned to the test set while the rest were used for model training (*n* = 209). This random split was done at the patient level, meaning there was no patient cross-over between the training and testing set which could represent a form of data leakage that could bias the results.

**Fig. 2:**
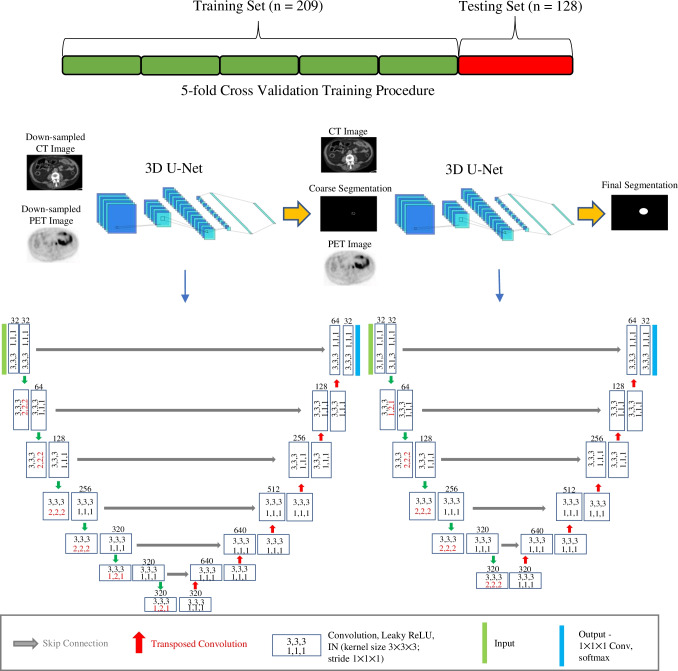
Schematic representation of the training procedure and overview of both U-Nets in the developed cascade architecture. Transposed convolution kernel and stride sizes in the decoder are equal to the stride size in the identical encoder resolution depth, highlighted in red in the architecture diagram. Feature map depths at each resolution are displayed above the convolution blocks and are capped at 320 regardless of the number of encoder-decoder stages. The output of the first 3D U-Net is upsampled and combined with the full resolution PET and CT images as an input to the second 3D U-Net

A 3D U-Net cascade, consisting of two 3D U-Nets, was trained using the nnU-Net self-configuring pipeline. Prior to input into the cascade network, patient CT scans were resampled into the same coordinate space as the PET images using B-spline interpolation and PET scans were converted into SUV_bw_. The first 3D U-Net in the cascade was trained on down-sampled PET and CT images (patch size = 80 × 80 × 224, voxel resolution = 5.22 × 5.22 × 2 mm^3^), so as to incorporate more contextual information from the images, and generated a coarse segmentation map. This segmentation map then served as a third channel input into the second 3D U-Net which was trained on full resolution images (patch size = 96 × 96 × 256, voxel resolution = 4.07 × 4.07 × 2 mm^3^) and yielded the final volumetric segmentation. Details of the cascade network and training procedure are shown in Fig. 2.

The U-Nets configured by the nnU-Net framework share much of the same characteristics as the original U-Net design [23], with minor modifications such as the use of instance normalisation, and leaky ReLU as the activation function. Both components of the cascade network were trained using five-fold cross-validation, where each fold was trained for a total of 1000 epochs using stochastic gradient descent with an initial learning rate of 0.01 that decayed to zero at the last training epoch. The dice and binary cross entropy loss functions were summed together with an equal weighting to create the final loss function used throughout training [37]. Further details about the nnU-Net design choices and empirical pipeline configurations based on dataset properties can be found in [30] and the associated GitHub repository.^1^ Models were trained on an NVIDIA Titan RTX GPU on PyTorch version 1.10.

### Model evaluation

The performance of the developed segmentation pipeline was assessed by way of voxel-level comparisons between the generated fully automatic segmentation mask output with the corresponding ground truth manual segmentation for each testing set scan. For a robust assessment of the network performance, evaluation metrics were calculated at three different levels, encapsulating the ability of the model to perform three different computer vision tasks, namely (i) patient-level scan classification, (ii) lesion-level detection, and (iii) voxel-level segmentation.

Patient-level scan classification refers to the ability of the model to correctly predict the PSMA-positivity status of the scan. A PSMA-positive scan was defined in this study as a PET/CT image in which at least one PSMA-avid lesion was detected in the ground truth manual segmentations. The criteria for a true positive scan prediction from the network was that at least one of the lesions in the PSMA-positive scan was detected, where detection in this instance was defined as having a volumetric overlap of at least 10% between the model output and the ground truth lesion delineation. The criterion for a true negative (TN) scan prediction from the model was that the network must not predict even a single positive voxel in that PSMA-negative scan. Utilising these criteria, classification performance was assessed by calculating the accuracy, sensitivity, PPV, specificity, and negative predictive value (NPV).

Lesion-level detection defines the ability of the model to detect the metastatic lesions identified in the ground truth delineations. As in the task of scan classification described above, a lesion was considered detected if at least 10% of the ground truth lesion volume was correctly predicted by the network. Contiguous and positive voxel clusters predicted by the network falling outside the ground truth lesion boundaries were counted and considered false positives. Detection performance was quantified by the calculation of the PPV, sensitivity, and F1 score, where the F1 score is defined as the harmonic mean of the PPV and sensitivity.

Network segmentation accuracy was assessed by voxel-level comparison of the automated model output with the ground truth contour, quantified through the Dice similarity coefficient (DSC), sensitivity, and PPV. For the 28 scans that received a second observer delineation (20 PSMA-positive, 8 PSMA-negative controls), patient-level, lesion-level, and voxel-level metrics were also calculated using the same criteria as described above, allowing the results of the model to be placed in the context of inter-observer variability.

### Fully automated biomarkers

Quantitative imaging biomarkers were extracted from the automatically generated segmentations and assessed for their potential to stratify patients based on overall survival (OS). Total lesional volume (TLV_auto_) was quantified by adding the volume of all positive voxels identified in the automated segmentations, and total lesional uptake (TLU_auto_) calculated by summing the SUVs of the identified positive voxels.

### Statistical analysis

Patient characteristics between the training and testing sets (age, PSA at referral for PSMA scan) were compared using a one-way analysis of variance (ANOVA). Stratification of patient survival based on calculated biomarkers from the automated model predictions was assessed using Kaplan–Meier analysis with the log rank test. Wilcoxon signed-rank tests were used to compare distribution of manual vs. automated biomarker calculations, and to compare the performance metrics between the automated model and second observer in the inter-observer analysis. Spearman correlation coefficient was used to assess the correlation between manual and automated biomarkers. In all cases, *p* < 0.05 was considered to be a statistically significant difference. Statistical analysis was performed in Python 3.7, using SciPy version 1.7.3 and Lifelines version 0.26.4.

## Results

### Characteristics of patients

The characteristics of the patients in the training and testing sets are summarised in Table 1. For both age and PSA values, no statistically significant difference was observed between the training and the testing set (both *p* > 0.05). Patient Gleason scores in both the training set and testing set had medians of 7 (training set range: 6–10; testing set range: 5–10). To facilitate survival analysis, testing set patients were followed up from the time of baseline scan until censoring date or death, with a median follow-up time of 71.5 months (range: 21.3–79.7 months).

**Table 1:** Characteristics of the patients in the training and testing set

Characteristic	Dataset		*p*-value
	Training set	Testing set	
No. of patients	121	72	
No. of scans	209	128	
No. of PSMA-positive scans (%)	209 (100%)	75 (58.6%)	
No. of PSMA-negative scans (%)	-	53 (41.4%)	
Age (mean ± SD, years)	70.4 ± 8.4	68.9 ± 7.1	0.218
PSA at scan referral (median, range, ng/mL)	3.50 [0.20, 79.46]	1.85 [0.20, 36.00]	0.255
Gleason score (median, range)	7 [6, 10]	7 [5, 10]	
No. of lesions	880	307	
Local prostate (*n*, % total)	141 (16.02%)	40 (13.03%)	
Regional nodal (*n*, % total)	191 (21.70%)	60 (19.54%)	
Distant nodal (*n*, % total)	356 (40.45%)	151 (49.19%)	
Osseous (*n*, % total)	167 (18.98%)	55 (17.92%)	
Visceral (*n*, % total)	25 (2.84%)	1 (0.33%)	

### Classification and detection performance

**Fig. 3:**
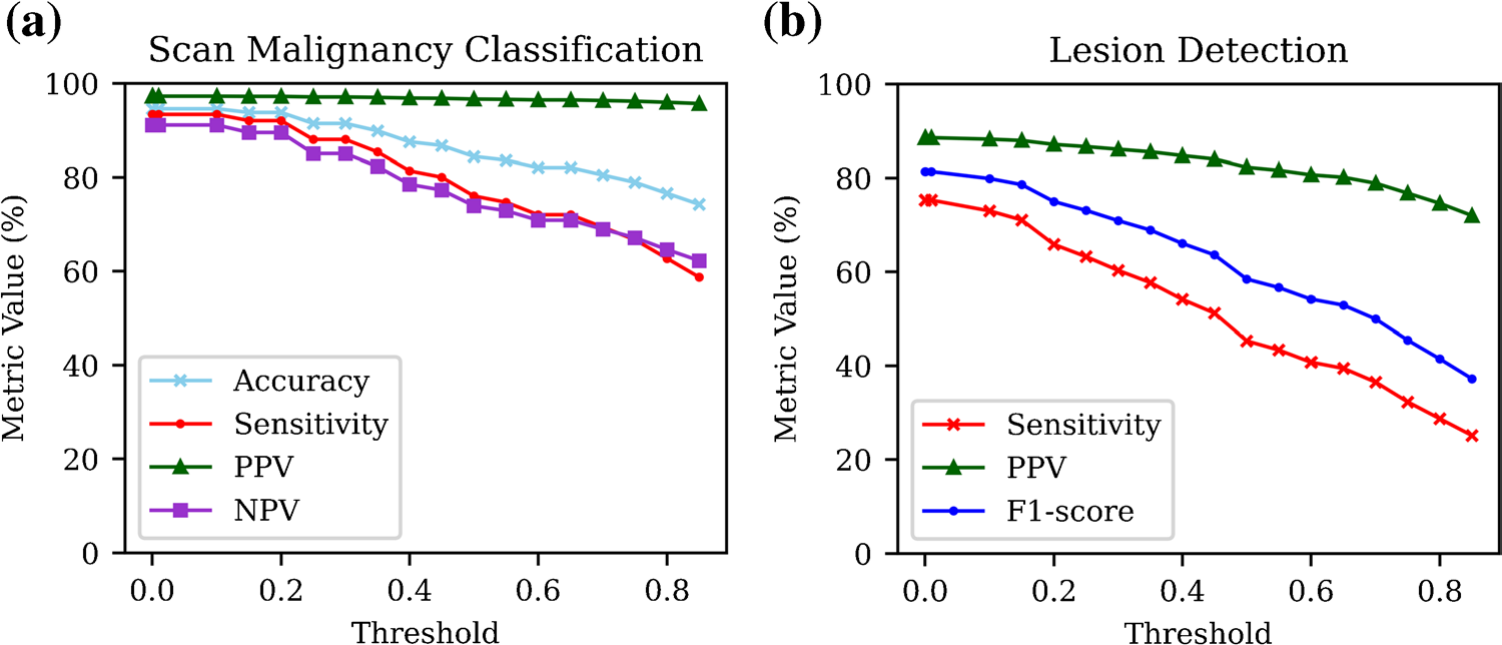
Plots showing the change in the calculated metrics as the threshold for a true positive is modified, for the tasks of (**a**) whole-scan malignancy classification and (**b**) individual lesion detection

The patient-level classification and lesion-level detection performance of the developed model are shown in Table 2. Whole scan classification performance as quantified by the accuracy, sensitivity, PPV, specificity, and NPV was > 90% in each case, with the model performing the best with respect to PPV (70/72, 97.2%), demonstrating a low false positive prediction rate in the PSMA-negative scans. Figure 3a shows the change in the classification performance metrics as the 10% volume overlap threshold criteria for a true positive prediction is modified.

**Table 2:** Fully automated model performance at all levels (patient level classification through to voxel-level segmentation) calculated on the dedicated test set

Task	Metric	Value
Patient-level classification	Accuracy (%)	94.5 (121/128)
	Sensitivity (%)	93.3 (70/75)
	PPV (%)	97.2 (70/72)
	Specificity (%)	96.2 (51/53)
	NPV (%)	91.1 (51/56)
Lesion-level detection	PPV (%)	88.2 (224/254)
	Sensitivity (%)	73.0 (224/307)
	F1 score (%)	79.9
Lesion sub-groups detection		
﻿Local prostate	Sensitivity (%)	90.0 (36/40)
Regional nodal	Sensitivity (%)	68.3 (41/60)
Distant nodal	Sensitivity (%)	76.2 (115/151)
Osseous	Sensitivity (%)	58.2 (32/55)
Visceral	Sensitivity (%)	0 (0/1)
Voxel-level segmentation	DSC (mean ± SD)	43.5 ± 21.5
	Sensitivity (mean ± SD)	45.0 ± 29.2
	PPV (mean ± SD)	58.5 ± 28.2

In the 128 [^68^Ga]Ga-PSMA-11 PET/CT scans in the testing dataset, a total of 307 lesions were identified in the ground truth manual delineations. Of these 307 lesions, 224 were correctly identified by the automated network, yielding a sensitivity of 73%. The calculated PPV of the model predictions was 88.2% (224/254). The automated model therefore yielded a total of 30 false positive lesion predictions across the 128 testing set scans, amounting to 1 false positive prediction every 4.3 patient scans. The 75 PSMA-positive scans contained 28 of these false positives (1 every 2.68 scans), while the 53 PSMA-negative scans contained only 2 of the false positive predictions (1 every 26.5 scans). Figure 3b shows the change in lesion detection metrics as the volume overlap threshold criteria of 10% is modified.

### Segmentation performance

**Fig. 4:**
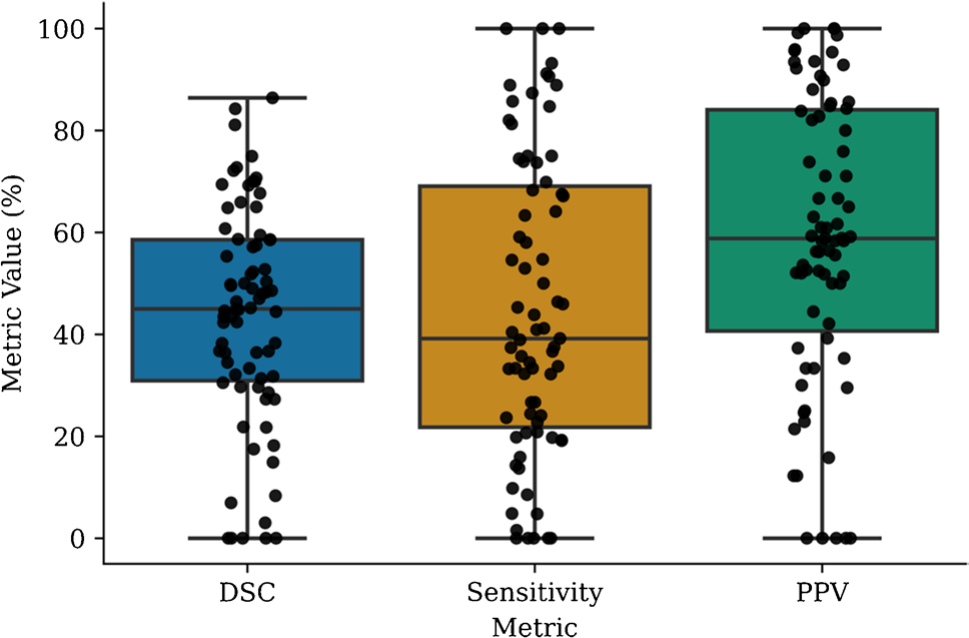
Distribution of the DSC, sensitivity, and PPV metrics calculated on all patient scans in the dedicated test set. Each dot represents the metric calculated at the voxel level for a whole patient scan

Voxel-level segmentation performance metrics are summarised in Table 2. The automated approach yielded mean DSC, sensitivity, PPV, and specificity values of 43.5%, 45%, 58.5%, and 99.9%, respectively. Boxplot distributions of the metrics for each testing set scan are presented in Fig. 4.

**Fig. 5:**
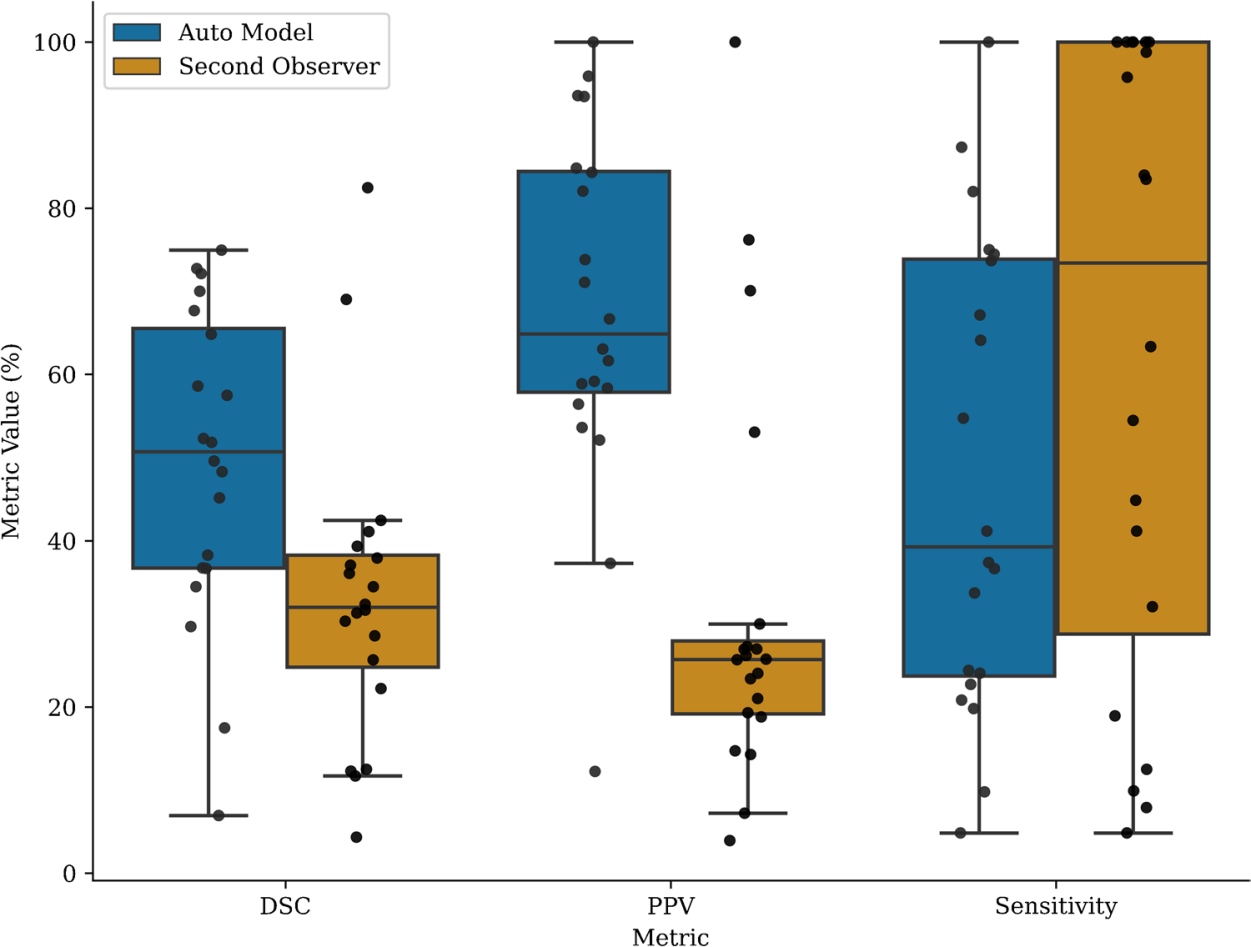
Distribution of the DSC, PPV, and sensitivity metrics calculated at the voxel level for both the automated model and observer 2 with respect to observer 1, showing how the model performs relative to inter-observer variability

Voxel-level comparisons of the automated segmentations with the second observer delineations are presented in Fig. 5. The DSC of the automated model was found to be significantly greater than the second observer DSC (median of 50.7% vs. 32%, *p* = 0.012). The PPV of the automated model was also found to be significantly greater than that of the second observer (median of 64.9% vs. 25.7%, *p* < 0.005). The sensitivity of the second observer was found to be greater than the developed model, but this difference did not reach statistical significance (median of 73.4% vs. 39.2%, *p* = 0.068). A complete comparison of the automated model and observer 2 with respect to observer 1 at all assessment levels (patient, lesion, and voxel) is presented in Table 3.

**Table 3:** Patient-level, lesion-level, and voxel-level results comparison between the automated model and observer 2 measured with respect to the observer 1 segmentations

Task	Metric	Automated model	Observer 2
Patient-level classification	Accuracy (%)	100 (28/28)	93.9 (26/28)
	Sensitivity (%)	100 (20/20)	100 (20/20)
	PPV (%)	100 (20/20)	90.9 (20/22)
	Specificity (%)	100 (8/8)	75 (6/8)
	NPV (%)	100 (8/8)	100 (6/6)
Lesion-level detection	PPV (%)	95.5 (63/66)	91.7 (66/72)
	Sensitivity (%)	68.5 (63/92)	71.7 (66/92)
	F1 score (%)	79.7	80.5
Lesion sub-groups detection			
Local prostate	Sensitivity (%)	100 (15/15)	93.3 (14/15)
Regional nodal	Sensitivity (%)	42.1 (8/19)	73.7 (14/19)
Distant nodal	Sensitivity (%)	62.8 (27/43)	62.8 (27/43)
Osseous	Sensitivity (%)	92.9 (13/14)	78.6 (11/14)
Visceral	Sensitivity (%)	0 (0/1)	0 (0/1)
Voxel-level segmentation	DSC (mean ± SD)	49.3 ± 18.9	33.1 ± 18.2
	Sensitivity (mean ± SD)	47.7 ± 28.4	62.6 ± 37.7
	PPV (mean ± SD)	67.9 ± 21.6	31.7 ± 24.3

### Automated vs. manual biomarkers

**Fig. 6:**
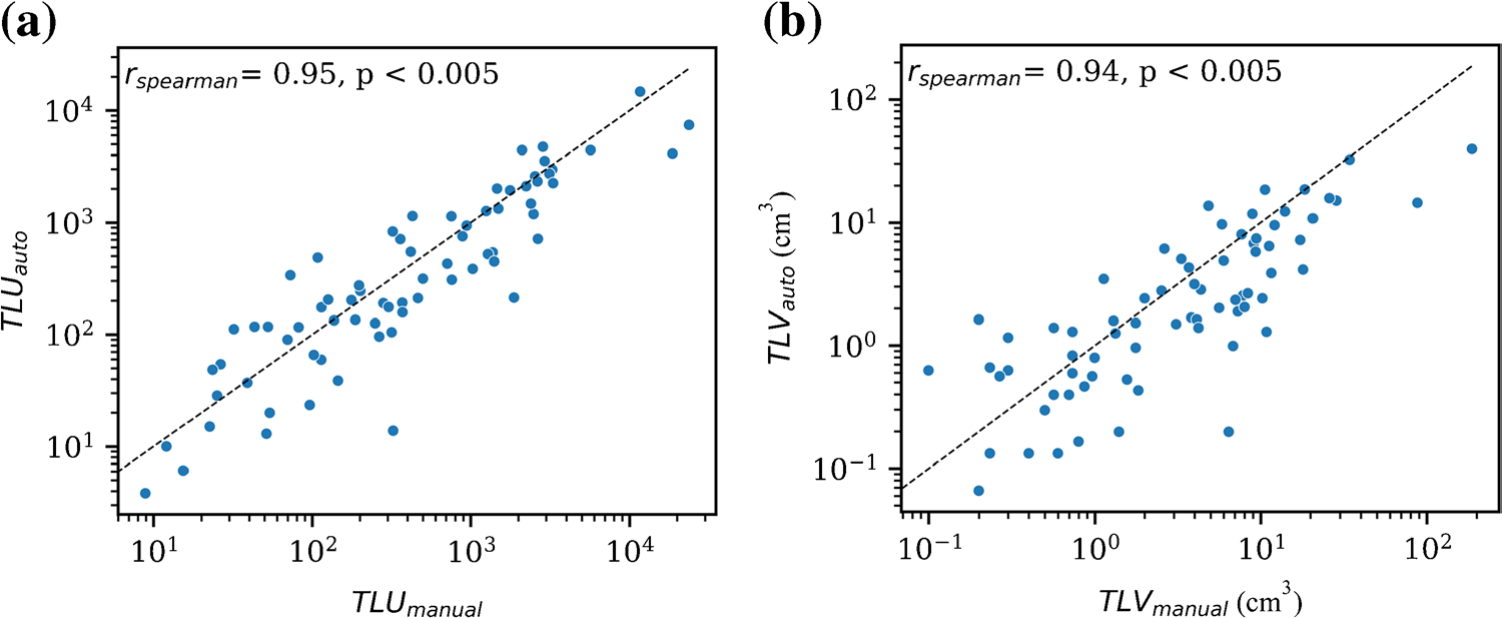
Scatter correlation plots between the manual and automatically calculated biomarkers on the test set scans. Strong positive correlations exist for both TLU_auto_ (*r*_spearman_ = 0.95, *p* < 0.005) and TLV_auto_ (*r*_spearman_ = 0.94, *p* < 0.005) and their manually derived counterparts. Blacked dashed lines in the plots represent the identity line. Axes have been log-transformed for better visual interpretation

Manual total uptake measurements, TLU_manual_, differed significantly from the fully automated total uptake measurements, TLU_auto_, with a tendency for the automated model to underestimate the uptake (median of 40.93 vs. 32.83, respectively, *p* = 0.049), and a strong positive correlation between the two was found (*r*_spearman_ = 0.95, *p* < 0.005). Similarly, the TLV_auto_ was found to be significantly different from TLV_manual_, with the automated model underestimating the volume (median of 0.398 cm^3^ vs. 0.43 cm^3^, respectively, *p* < 0.005). A strong positive correlation between the two measurements was found (*r*_spearman_ = 0.94, *p* < 0.005). Correlation results are presented in Fig. 6.

### Automated biomarkers and overall survival

**Fig. 7:**
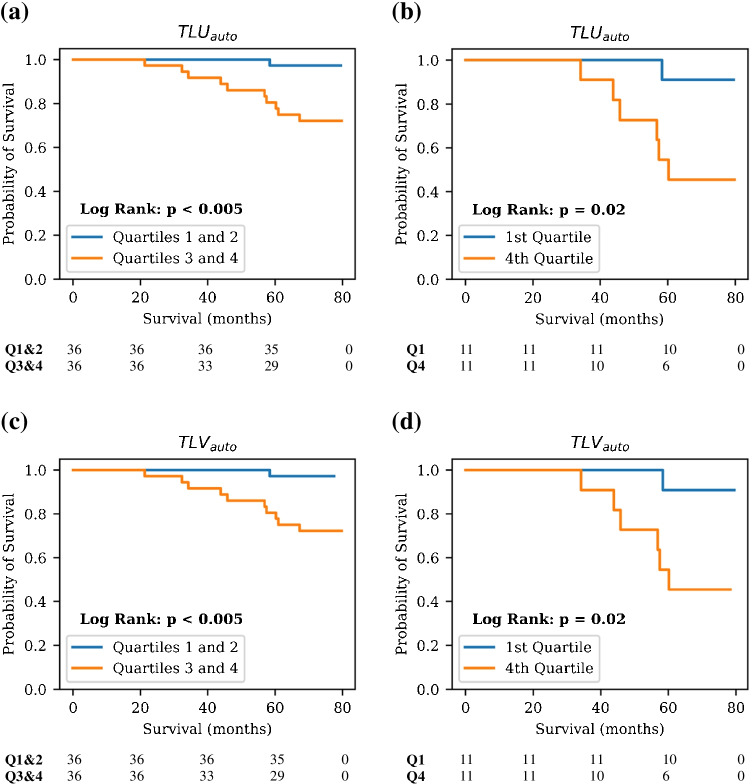
Kaplan–Meier analyses on the calculated biomarkers from the test set segmentations. Patient baseline scans only are included. Plots are shown for TLU_auto_ calculated for: (**a**) the entire test set and (**b**) just the PSMA-positive scans in the test set, and for TLV_auto_ calculated on (**c**) the whole test set, and (**d**) just the PSMA-positive scans. Number of patients still at risk for both groups, defined as patients that have not experienced the outcome of interest and have not been censored, are included below each plot

**Fig. 8:**
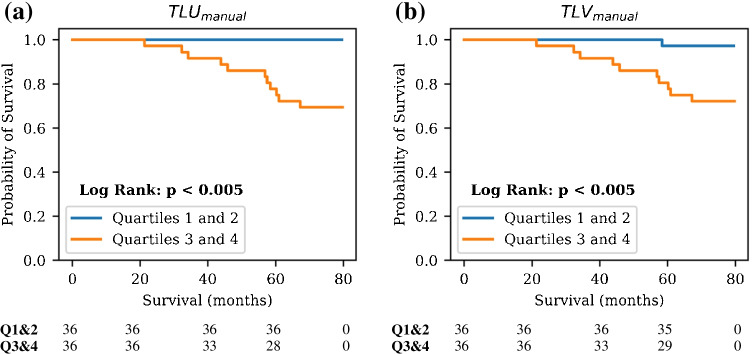
Kaplan–Meier plots on the (**a**) total lesional uptake and (**b**) total lesional volume calculated from the ground truth manual contours in the whole testing set PSMA scans. Patient baseline scans only are included in the analysis. Number of patients still at risk, defined as patients that have not experienced the outcome of interest and have not been censored, are included below each plot

Upon stratification of the testing set patients based on the median TLU_auto_ value, a statistically significant difference in OS was detected between the two groups (*p* < 0.005). Due to the potential for the PSMA-negative scans in the testing set to influence the results, stratification was also performed on just the PSMA-positive scans between quartile 1 and quartile 4 of TLU_auto_, and a significant difference in OS was found between the two groups (*p* = 0.02). Similarly, patient stratification according to median TLV_auto_ yielded a statistically significant difference between the two groups in OS (*p* < 0.005). Considering only PSMA-positive test scans, quartile 1 and quartile 4 stratification resulted in a statistically significant difference in OS (*p* = 0.02). Graphical results are presented in Fig. 7. Kaplan–Meier analyses conducted on the ground truth manual contours in the whole testing set are also provided in Fig. 8.

## Discussion

PSMA-targeted imaging is emerging as the standard of care in the diagnostic workup of PCa patients. The fully automatic assessment of whole-body PSMA PET scans has the potential to streamline clinical workflows, alleviate the inter-observer variability inherent in manual and semi-automatic mPCa lesion detection and segmentation, and lay the groundwork for the generation of unique quantitative biomarkers with prognostic potential, perhaps ultimately paving the way towards a truly precision medicine–based approach to the management of patients. In this study, a fully automated mPCa lesion segmentation model was developed for whole-body [^68^Ga]Ga-PSMA-11 PET/CT images using the self-configuring nnU-net framework. The predictions of the dual-channel network, incorporating hybrid information from both PET and CT images, were assessed on a dedicated test set with respect to numerous tasks, including whole-scan malignancy classification, lesion detection, voxel-level segmentation, and ability to generate useful biomarkers significantly associated with patient OS. The results achieved demonstrate the feasibility of automated whole-body PSMA scan assessment using deep learning.

Staging of prostate cancer in the form of the TNM criteria remains an important prognostic tool in the clinical management of the disease [38]. The classification of the malignancy of patient scans is therefore of clinical importance. The developed model in this work was able to classify the malignancy status of patient scans with a very high degree of accuracy (> 90% with respect to all calculated metrics) in a testing set containing both PSMA-negative controls and PSMA-positive images, indicating a potential clinical use for the automated model. These results were comparable to a similar study conducted by Liu et al. [32], who built a 3D cascade U-Net for both bone segmentation and bone metastasis segmentation. Their two-step model achieved sensitivity of 93.6% and specificity of 93.8% in predicting the M-staging from mpMRI scans in the pelvic area (the present work yielded sensitivity of 93.3%, and specificity of 96.2%). The authors had noted that extension of their analyses to whole-body scans was a necessary condition for clinical implementation. The present study demonstrates that this can be done on PSMA PET/CT scans with comparable accuracy, and without restricting the analysis to just bone lesions.

Distinguishing physiologic PSMA-ligand uptake from pathologic uptake poses a challenge in the implementation of segmentation techniques for PSMA scans. Semi-automated approaches require manual user input to remove physiologic uptake areas, through either manual adjustment of the segmentation mask or the deletion of the mask entirely [33, 34]. If global thresholding techniques are used in the semi-automated process, false positives may be numerous and require significant manual intervention to remove. The developed model in this work yielded only 30 false positive lesion predictions across the entire testing cohort of 128 scans, amounting to 1 false positive prediction every 4.3 patient scans. This is less than that of a recent study by Trägårdh et al. [35], whose AI model for detection of pelvic lymph node metastases in [^18^F]PSMA PET/CT scans yielded 1.8 false positive predictions per patient.

The great performance of our model in terms of low false positive predictions comes at the expense of model sensitivity. The overall lesion-level sensitivity of 73% is lower than the Trägårdh et al. study [35] average sensitivity of 82% and another work by Zhao et al. [31] who achieved sensitivity of more than 90% overall for pelvic area mPCa lesion detection in [^68^Ga]Ga-PSMA-11 scans. However, both of these works developed AI models for lesion detection exclusively in the pelvic area — our model extends this to whole-body mPCa lesion detection, a more difficult task. Furthermore, in the interobserver analysis, our AI model lesion sensitivity is comparable to that of the second observer (68.5% vs. 71.7%). We are currently investigating techniques to improve the model sensitivity, such as re-training with the Tversky loss function which can be used to prioritise model sensitivity [39], and potentially training multiple models for different lesion types (e.g., one model for bone lesions, another for nodal metastases). This could particularly help with improving the detection of bone lesions (58.2% sensitivity), where specific image pre-processing techniques such as CT Hounsfield unit bone thresholding can be implemented to aid the model in localising this lesion type.

Relative to classification and detection, performance metrics for voxel-level segmentation (the most difficult of the three tasks) were low. The myriad of different lesion sizes, uptake values, and anatomical locations that the model needs to segment throughout the whole-body scan is a likely contributing factor. The number of metastases available for model training could also have contributed — we had less lesions available for training than the Zhao et al. study [31]. The performance of our model can doubtless be improved through the addition of more high-quality training data from which the model can learn. Importantly, however, it was shown that for the subset of scans that received a second observer delineation, the DSC and the PPV were found to be significantly greater for the automated model compared with observer 2 (*p* = 0.012 and *p* < 0.005, respectively). Sensitivity of the model was inferior to observer 2, but this did not reach the level of statistical significance (*p* = 0.068). Therefore, as measured by the DSC and the PPV, the automated model performs within the measured interobserver variability. Furthermore, the voxel-level lesion predictions enable the extraction of features from images with prognostic information that can inform clinical decision-making. Numerous studies have demonstrated that quantitative biomarkers derived from PSMA-PET images, typically quantified through semi-automated segmentation approaches, have significant prognostic potential in the management of mPCa patients [33, 40–42]. In this study, we conducted an additional validation of our automated model by demonstrating that several global biomarkers derived from the fully automatic lesion segmentations, TLU_auto_ and TLV_auto_, were able to stratify patients based on OS with a Kaplan–Meier analysis to statistical significance. Fully automatic calculation of tumour burden metrics can be used for fast identification of high-risk patients and has advantages over manual and semi-automatic techniques which still require some measure of manual user input and are therefore susceptible to inter- and intra-observer variability.

Fully automated voxel-level segmentations also lay the groundwork for the high-throughput extraction of quantitative features at both the lesion level and the patient level. This radiomics approach to mPCa characterisation has demonstrated significant diagnostic and prognostic potential in the management of the disease [16, 19, 43, 44]. A crucial part of the radiomics workflow is the segmentation of regions of interest from which the quantitative features are extracted, however, the inter-observer variability of manual delineations is known to introduce a bias into this aspect of the workflow that can affect the resulting feature calculation [45]. The deterministic nature of the automated model developed in this work can mitigate this bias — the same scan inputted into the network multiple times will yield the same segmentation result each time, potentially increasing feature reproducibility. A detailed study of individual lesion-level radiomics features extracted from fully automated segmentations was out of the scope of the present study, however, and is recommended for future investigations.

It must be noted that the acquisition of patient data from a single institution in the present work can lead to a risk of selection bias — multicentre studies with larger patient cohorts are required to fully elucidate the potential clinical benefit of this model. Furthermore, the manual delineations that were utilised as the ground truth to train the model cannot be considered as a perfect ground truth. In addition to having documented inter-observer variability, as demonstrated in this study, partial volume effects in the PSMA-PET image can introduce inaccuracies that cause the observed lesion outline in the image to differ from the real pathologic lesion boundary [46]. Obtaining precise histopathologic boundaries for mPCa patients, who may have high numbers of metastatic lesions in diverse anatomical locations, is of course impractical, and thus manual annotation is used in this study as an approximation to the ground truth. It is also important to note that this particular cohort of PCa patients were either negative or oligometastatic on conventional staging imaging (bone scintigraphy scan with CT), and as a result there is a possibility that the dataset was biased towards patients with low disease burdens. Further validation of the model in higher disease burden populations is warranted.

## Conclusion

In this study, the feasibility of using deep learning techniques for the automated segmentation of mPCa lesions in whole-body [^68^Ga]Ga-PSMA-11 PET/CT scans to automatically extract patient-level prognostic biomarkers was investigated. The malignancy of patient scans was classified to a high degree of accuracy, and voxel-level segmentations as measured by the DSC and PPV performed within measured inter-observer variability. Biomarkers extracted from the automated segmentations (TLU_auto_ and TLV_auto_) also showed significant univariate association with patient overall survival. Multicentre studies with larger patient cohorts are required to confirm these promising findings.

## Footnotes

1 https://github.com/MIC-DKFZ/nnUNet
